# Enhancing therapeutic potential: Human adipose‐derived mesenchymal stem cells modified with recombinant adeno‐associated virus expressing VEGF165 gene for peripheral nerve injury

**DOI:** 10.1002/kjm2.12875

**Published:** 2024-08-05

**Authors:** Shuai Jiang, Bo Chen, Zhen‐Yu Sun

**Affiliations:** ^1^ Department of Orthopedics, The First Affiliated Hospital, College of Medicine Zhejiang University Zhejiang Hangzhou China

**Keywords:** mesenchymal stem cells, nervous system diseases, peripheral nerve injuries, recombinant adeno‐associated viruses, vascular endothelial growth factor A

## Abstract

This study aimed to investigate the therapeutic potential of human adipose‐derived mesenchymal stem cells (hADSCs) modified with recombinant adeno‐associated virus (rAAV) carrying the vascular endothelial growth factor 165 (VEGF165) gene in peripheral nerve injury (PNI). The hADSCs were categorized into blank, control (transduced with rAAV control vector), and VEGF165 (transduced with rAAV VEGF165 vector) groups. Subsequently, Schwann cell differentiation was induced, and Schwann cell markers were assessed. The sciatic nerve injury mouse model received injections of phosphate‐buffered saline (PBS group), PBS containing hADSCs (hADSCs group), rAAV control vector (control‐hADSCs group), or rAAV VEGF165 vector (VEGF165‐hADSCs group) into the nerve defect site. Motor function recovery, evaluated through the sciatic function index (SFI), and nerve regeneration, assessed via toluidine blue staining along with scrutiny of Schwann cell markers and neurotrophic factors, were conducted. Modified hADSCs exhibited enhanced Schwann cell differentiation and elevated expression of Schwann cell markers [S100 calcium‐binding protein B (S100B), NGF receptor (NGFR), and glial fibrillary acidic protein (GFAP)]. Mice in the VEGF165‐hADSCs group demonstrated improved motor function recovery compared to those in the other three groups, accompanied by increased fiber diameter, axon diameter, and myelin thickness, as well as elevated expression of Schwann cell markers (S100B, NGFR, and GFAP) and neurotrophic factors [mature brain‐derived neurotrophic factor (BDNF) and glial cell‐derived neurotrophic factor (GDNF)] in the distal nerve segment. rAAV‐VEGF165 modification enhances hADSC potential in PNI, promoting motor recovery and nerve regeneration. Elevated Schwann cell markers and neurotrophic factors underscore therapy benefits, providing insights for nerve injury strategies.

## 1 INTRODUCTION

Peripheral nerve injury (PNI) stands as a formidable global clinical and socioeconomic challenge, with autologous nerve anastomosis currently established as the gold standard for its treatment.^1^ Epidemiological studies in humans have disclosed an annual incidence rate of 13.9 individuals per 100,000 habitants for peripheral nervous system (PNS) injuries, and 2%–5% of patients admitted to level I trauma centers are potentially susceptible to PNS injuries.^2^ Despite the routine performance of nerve surgery, achieving satisfactory functional recovery remains a challenge, with only 40%–50% of individuals undergoing peripheral nerve surgery experiencing optimal outcomes.^3^ These circumstances underscore the imperative for innovative therapeutic strategies.

Schwann cells, integral myelinating cells of the PNS, play a pivotal role in supporting nerve regeneration by fostering axonal sprouts and facilitating the reinnervation of distal structures.^4^ The remarkable ability of Schwann cells to revert to a repair‐competent state significantly contributes to the intricate regeneration process following PNI.^5^ Adipose‐derived stem cells (ADSCs), a subtype of mesenchymal stem cells, offer a promising alternative due to their multipotent capabilities, immunomodulatory properties, and tissue regeneration potential through paracrine actions via extracellular vesicles containing trophic factors.^6^ Easily obtained through low‐invasive procedures, ADSCs present a readily harvestable and rapidly proliferating substitute with immune defense resilience, making them an attractive candidate for nerve regeneration therapies.^7^

The heightened brain activity induced by transplanted human hADSCs likely results from their secretion of various growth factors (GFs) and neurotrophic factors (NFs) following PNI, including brain‐derived neurotrophic factor (BDNF) and glial‐derived neurotrophic factor (GDNF).^8^ BDNF, initially identified for its role in supporting sensory neurons, is now recognized for its multifaceted trophic functions.^9^ Studies suggest that BDNF produced by bone marrow‐derived mesenchymal stem cells (BMSCs) synergistically promotes peripheral nerve repair in vivo.^10^ GDNF, first isolated and purified from the conditioned medium of the mouse glial cell line B49,^11^ primarily interacts with glycosylphosphatidylinositol‐linked GDNF (GFRα) and the tyrosine kinase rearranged during transfection (RET) receptor to support neuronal development, survival, and regeneration across both the central and peripheral nervous systems.^12^ GDNF is widely distributed in the PNS and various cell types, including glial cells such as astrocytes, oligodendrocytes, and Schwann cells, as well as neurons such as motor, enteric, sympathetic, and dopaminergic neurons, and target tissues such as skeletal muscle.^13^

The vascular endothelial growth factor (VEGF) family encompasses five glycoproteins (VEGF‐A, VEGF‐B, VEGF‐C, VEGF‐D, and VEGF‐E) with six isoforms (121, 145, 165, 183, 189, and 206 amino acids) generated through alternative splicing.^14^ VEGF‐A comprises the isoforms VEGF121, VEGF165, and VEGF189, secreted by various cell types and functioning as disulfide‐linked homodimers.^15^ In vitro studies on primary Schwann cell cultures have demonstrated that VEGF165 stimulation increases Schwann cell migration, a pivotal process in promoting neurite outgrowth.^16^ Genetic modification of hADSCs using recombinant adeno‐associated virus (rAAV) carrying the VEGF165 gene has shown enhanced VEGF secretion, indicating significant therapeutic effects in promoting angiogenesis with potential clinical applications.^17^

This study aims to unravel the therapeutic potential of hADSCs modified with rAAV‐VEGF165 in the context of PNI. By evaluating the impact of VEGF165 gene transduction on Schwann cell differentiation and functional outcomes, as well as BDNF and GDNF expression in a sciatic nerve injury mouse model, we aim to contribute to innovative strategies for PNI treatment, potentially revolutionizing therapeutic approaches in this field.

## 2 METHODS AND MATERIALS

### 2.1 Ethics statement

All experimental procedures involving laboratory animals were approved by the Institutional Animal Care and Use Committee of our hospital, adhering to the Guidelines for the Care and Use of Laboratory Animals.^18^

### 2.2 Cell culture and grouping

The normal human ADSCs (hADSCs) (Catalog: PCS‐500‐011, ATCC, USA) are fibroblast‐like cells cultured in mesenchymal stem cell basal medium (Catalog: PCS‐500‐030, ATCC, USA) supplemented with a mesenchymal stem cell growth kit (Catalog: PCS‐500‐040, ATCC, USA). The growth supplements include MSC Supplement (2% FBS, 5 ng/mL rh FGF basic, 5 ng/mL rh FGF acidic, 5 ng/mL rh EGF) and 2.4 mM l‐alanyl‐l‐glutamine. The hADSCs at passage 3 were divided into three distinct groups: the blank group (no transduction), the control group (transduced with rAAV control), and the VEGF165 group (transduced with rAAV VEGF165). The cells underwent transduction using rAAV serotype 2 carrying the human VEGF165 gene or the rAAV control. To generate rAAV particles, the Hind3/EcoR1 fragment containing the human VEGF165 gene was excised from pcDNA3‐Hvegf. Subsequently, it was cloned with the “stuffer” EcoR1/Xho1 fragment of pCMV‐LUC‐KEB into the pAAV‐MCS vector (Catalog: 28252101, Applied Biological Materials Inc., Canada). The resulting construct, pAAV‐VEGF165, was generated by digestion with Hind3 and Xho1. The generation of rAAV particles occurred in HEK293T cells (Catalog: CRL‐1573, ATCC, USA) using the AAV Helper‐Free System (Catalog: 240071, Stratagene, USA). One day before transduction, hADSCs were seeded into 100‐mm plates at a concentration of 6 × 10^5^ cells/dish in culture medium. Infected cells were cultured for 48 h, and the infection efficiency was evaluated through fluorescence.

### 2.3 MTT assay

The cell viability of hADSCs was assessed using a 3‐(4,5‐dimethylthiazol‐2‐yl)‐2,5‐diphenyltetrazolium bromide (MTT) assay on days 1, 3, 5, and 7. Cells were treated with 0.5 mg/mL MTT reagent (Sigma Aldrich, St. Louis, Missouri, USA) for 2 h at 37°C in a 5% CO_2_ chamber. Following incubation, the MTT solution was removed, and an equal volume of isopropyl alcohol (Sigma–Aldrich, St. Louis, Missouri, USA) was added, followed by shaking at room temperature for 10 min. The absorbance of the resulting solutions was measured at 570 nm using an ELISA reader (Versamax, Molecular Devices, San Jose, California, USA).

### 2.4 Schwann cell induction

Gene‐modified hADSCs (passage 3) were seeded in duplicate on 6‐well plates and maintained in culture medium until reaching ~90% confluency. Subsequently, Schwann cell induction was performed. hADSCs underwent a 24‐h treatment with 1 mM β‐mercaptoethanol, followed by a 72‐h treatment with 35 ng/mL all‐trans retinoic acid. Both substances were dissolved in growth medium, composed of 90% minimum essential medium (α‐MEM), 2 nM l‐glutamine, and 10% fetal bovine serum. The cells were then differentiated for 14 days under specific differentiation conditions. The differentiation medium included growth medium supplemented with 5 ng/mL platelet‐derived growth factor, 10 ng/mL basic fibroblast growth factor, 14 mM forskolin, and 192 ng/mL glial growth factor 2. The medium was changed every 2–3 days. After 14 days, the morphology of hADSCs was observed using an inverted microscope. VEGF165 expression was evaluated through both qRT‐PCR and Western blotting in hADSCs and differentiated Schwann cells. Furthermore, the expression levels of Schwann cell markers (S100B, NGFR, and GFAP) were assessed using qRT‐PCR and Western blotting specifically in the differentiated Schwann cells.

### 2.5 In vivo experiments

Twenty‐four 8‐week‐old male BALB/c nude mice (20–25 g) were categorized into four groups: PBS, hADSCs, control‐hADSCs, and VEGF165‐hADSCs, each comprising six mice. Under isoflurane anesthesia, a 1 cm incision was made on the right hind limb after local skin disinfection. The biceps femoris muscle was gently separated to expose the right sciatic nerve. Following a previously described protocol,^1^ nerve injury was induced by cutting the central area, followed by immediate cell transplantation. hADSCs, hADSCs infected with rAAV VEGF165 vector, or hADSCs infected with AAV blank control vector were digested with trypsin and suspended in PBS. Subsequently, 100 μL of either PBS or PBS containing 1 × 10^6^ cells was directly injected into the nerve defect site of each mouse in the respective groups. After wound closure, footprint analysis was conducted weekly for a total of 4 weeks. The hind paws of mice were painted with red ink, and they were allowed to walk down a white paper‐covered track at 1‐, 2‐, 3‐, and 4‐weeks post‐surgery. Three mice in each group were assessed, with each mouse walking along the track three times. The hind paw prints were scanned, and parameters such as print length (PL), toe spread (TS), and intermediary toe spread (IT) were measured to calculate the sciatic function index (SFI).^19^ After footprint analysis, the mice were euthanized, and the dissected nerves were fixed and processed for further analysis. The nerves were fixed with 2.5% glutaraldehyde overnight at 4°C, postfixed with 1% osmium tetroxide (OsO4) for 2 h, dehydrated, and embedded in epoxy resin. Semi‐thin sections (1 μm) were stained with a 1% toluidine blue solution, and images were captured under a light microscope (Olympus IX‐73). Myelinated fiber analysis via ImageJ software from the National Institutes of Health (Bethesda, MD), included quantification of axon density per 10,000 μm^2^, alongside measurements of the outer and inner margins of the myelin sheath, which were utilized to calculate fiber diameter (FD) and axon diameter (AD), allowing for the determination of myelin thickness (FD–AD).^20^ Subsequently, qRT‐PCR and Western blotting were employed to assess the expression of Schwann cell markers and neurotrophic factors in the distal nerve segment.

### 2.6 Quantitative real‐time polymerase chain reaction (qRT‐PCR)

For qRT‐PCR analysis, total RNA was extracted from the collected samples using a Trizol Plus RNA purification kit (Catalog: 12183555, Thermo Fisher Scientific Inc., Shanghai, China). Complementary DNA (cDNA) synthesis was performed using a high‐capacity cDNA reverse transcription kit (Catalog: 4368814, Thermo Fisher Scientific Inc., Shanghai, China) with equal amounts of RNA from each sample. The resulting cDNA served as a template for qRT‐PCR, which was conducted using specific primers for the target gene (Table 1) and an SYBR Green PCR Master Mix (Catalog: 367659, Thermo Fisher Scientific Inc., Shanghai, China). The qRT‐PCR reactions were run on a thermal cycler, and the obtained data were analyzed using appropriate software. The relative gene expression levels were normalized to glyceraldehyde‐3‐phosphate dehydrogenase (GAPDH) and calculated using the 2^−ΔΔCT^ method.

**TABLE 1:** Primer sequences for quantitative reverse‐transcription polymerase chain reaction (qRT‐PCR).

Gene	Species	Primer	Sequence (5′→3′)
VEGF165	Human	Forward	GACAAGAAAATCCCTGTGG
		Reverse	TCAAGCTGCCTCGCCTTGCAACG
S100B	Human	Forward	TGGCCCTCATCGACGTTTTC
		Reverse	ATGTTCAAAGAACTCGTGGCA
NGFR	Human	Forward	CCTACGGCTACTACCAGGATG
		Reverse	CACACGGTGTTCTGCTTGT
GFAP	Human	Forward	CTGCGGCTCGATCAACTCA
		Reverse	TCCAGCGACTCAATCTTCCTC
GAPDH	Human	Forward	GGAGCGAGATCCCTCCAAAAT
		Reverse	GGCTGTTGTCATACTTCTCATGG
S100B	Mouse	Forward	TGGTTGCCCTCATTGATGTCT
		Reverse	CCCATCCCCATCTTCGTCC
NGFR	Mouse	Forward	CTAGGGGTGTCCTTTGGAGGT
		Reverse	CAGGGTTCACACACGGTCT
GFAP	Mouse	Forward	CCCTGGCTCGTGTGGATTT
		Reverse	GACCGATACCACTCCTCTGTC
BDNF	Mouse	Forward	TCATACTTCGGTTGCATGAAGG
		Reverse	AGACCTCTCGAACCTGCCC
GDNF	Mouse	Forward	TCATACTTCGGTTGCATGAAGG
		Reverse	AGACCTCTCGAACCTGCCC
GAPDH	Mouse	Forward	AGGTCGGTGTGAACGGATTTG
		Reverse	TGTAGACCATGTAGTTGAGGTCA

### 2.7 Western blotting

Protein extraction from the collected samples was performed using lysis buffer, and the protein concentration was determined using a Pierce™ BCA protein assay kits (Catalog: 23225, Thermo Fisher Scientific Inc., Shanghai, China). Equal amounts of protein from each sample were separated by sodium dodecyl sulfate‐polyacrylamide gel electrophoresis (SDS‐PAGE, Catalog: 89888, Thermo Fisher Scientific Inc., Shanghai, China) and then transferred to a polyvinylidene fluoride (PVDF, Catalog: 88585, Thermo Fisher Scientific Inc., Shanghai, China) membrane. After blocking, the membrane was incubated with primary antibodies specific to the target proteins, including VEGF 165 antibody at 1/1000 dilution (Catalog: ab106580), S100B antibody at 1/5000 dilution (Catalog: ab52642), NGFR antibody at 1/1000 dilution (Catalog: ab52987), GFAP antibody at 1/10000 dilution (Catalog: ab68428), mature BDNF antibody at 1/1000 dilution (Catalog: ab108319), GDNF antibody at 1/1000 dilution (Catalog: ab176564), and β‐actin antibody at 1/1000 dilution (Catalog: ab8227), all sourced from Abcam (USA). Subsequently, the membrane was probed with goat anti‐rabbit HRP at a 1/2000 dilution. Protein bands were visualized using an enhanced chemiluminescence (ECL) detection system (Catalog: WP20005, Thermo Fisher Scientific Inc., Shanghai, China), and protein expression levels were normalized to a loading control (β‐actin).

### 2.8 Statistical analysis

Statistical analysis was performed using GraphPad Prism 8.0 Software, and the results are presented as mean ± standard deviation (SD). In vitro experiments were analyzed using one‐way analysis of variance (ANOVA) followed by Holm–Sidak's multiple comparisons test to compare values among the three groups. For in vivo *e*xperiments, one‐way ANOVA followed by Holm–Sidak's multiple comparisons test was used for comparisons among four groups. The cell viability of hADSCs in vitro and the SFI in vivo were compared using a two‐way ANOVA followed by post‐hoc Tukey's multiple comparisons test for analysis. The significance level was set at *p* < 0.05.

## 3 RESULTS

### 3.1 VEGF165 overexpression facilitates the differentiation of hADSCs into Schwann cells

**FIGURE 1:**
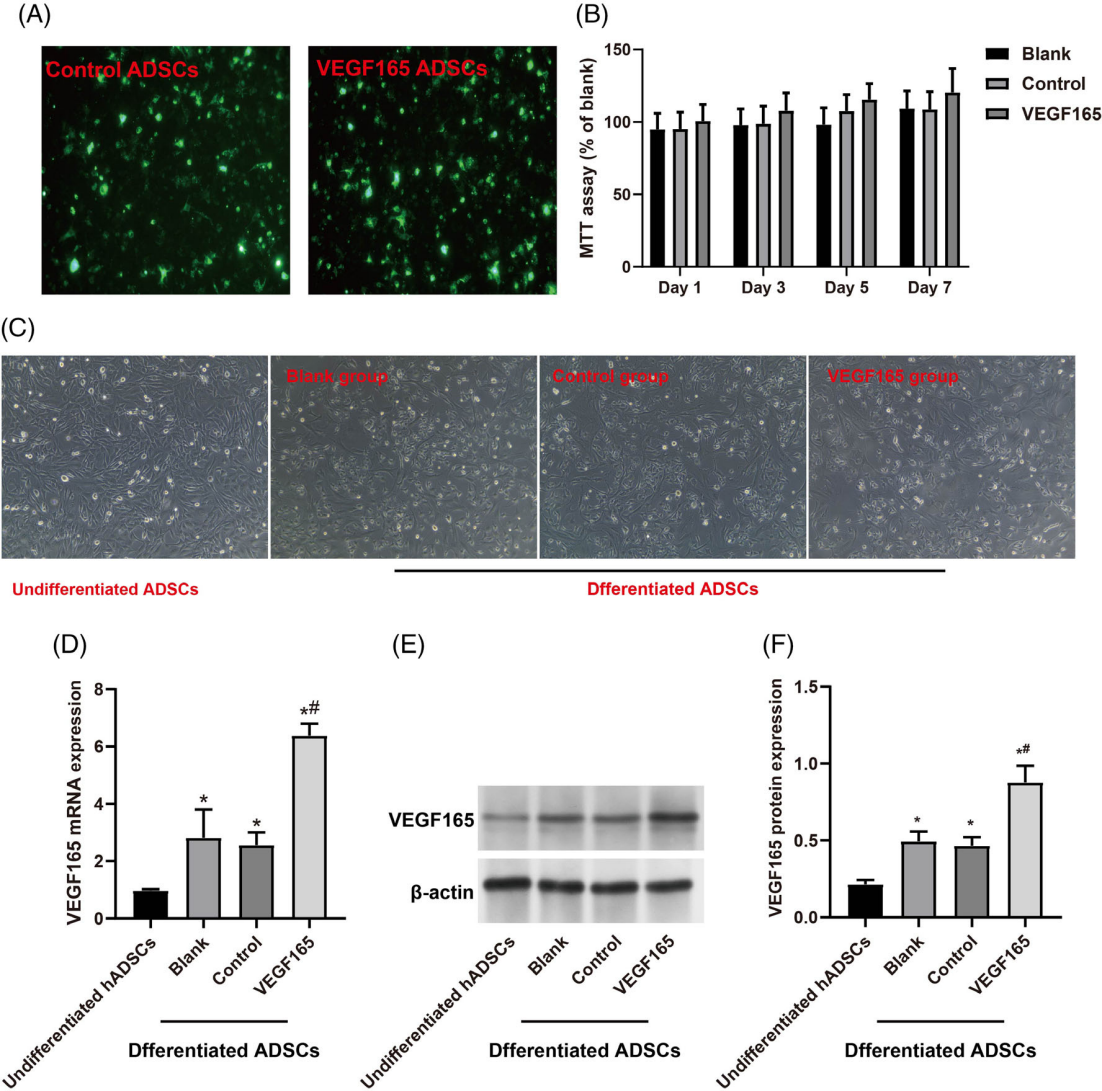
Recombinant adeno‐associated virus (rAAV)‐mediated enhancement of VEGF165 facilitates Schwann cell differentiation in hADSCs. (A) Distinct green fluorescence was observed within the cytoplasm of both control ADSCs and VEGF165 ADSCs after infection, indicating high infection efficiency. (B) Cell viability of hADSCs measured by the MTT assay, expressed as % viability compared to the blank group. (C) Representative inverted microscope image of hADSCs after 14 days of Schwann cell induction. Without induction, hADSCs exhibited a mesh‐like structure, while induced cells showed a spindle shape with reduced volume, fewer protrusions, and a spiral growth pattern resembling Schwann cells. Notably, the VEGF165 group exhibited more pronounced Schwann cell‐like features compared to the blank and control groups. (D–F) Analysis of VEGF165 gene and protein expression in undifferentiated and differentiated hADSCs by quantitative real‐time polymerase chain reaction (qRT‐PCR, D) and Western blotting (E,F), respectively. Experimental groups include the blank group (without infection), control group (rAAV control infection), and VEGF165 group (rAAV VEGF165 infection). Data are presented as the mean ± SD of three independent experiments. * indicates significance compared to undifferentiated hADSCs. # indicates significance compared to the blank and control groups at *p* < 0.05.

**FIGURE 2:**
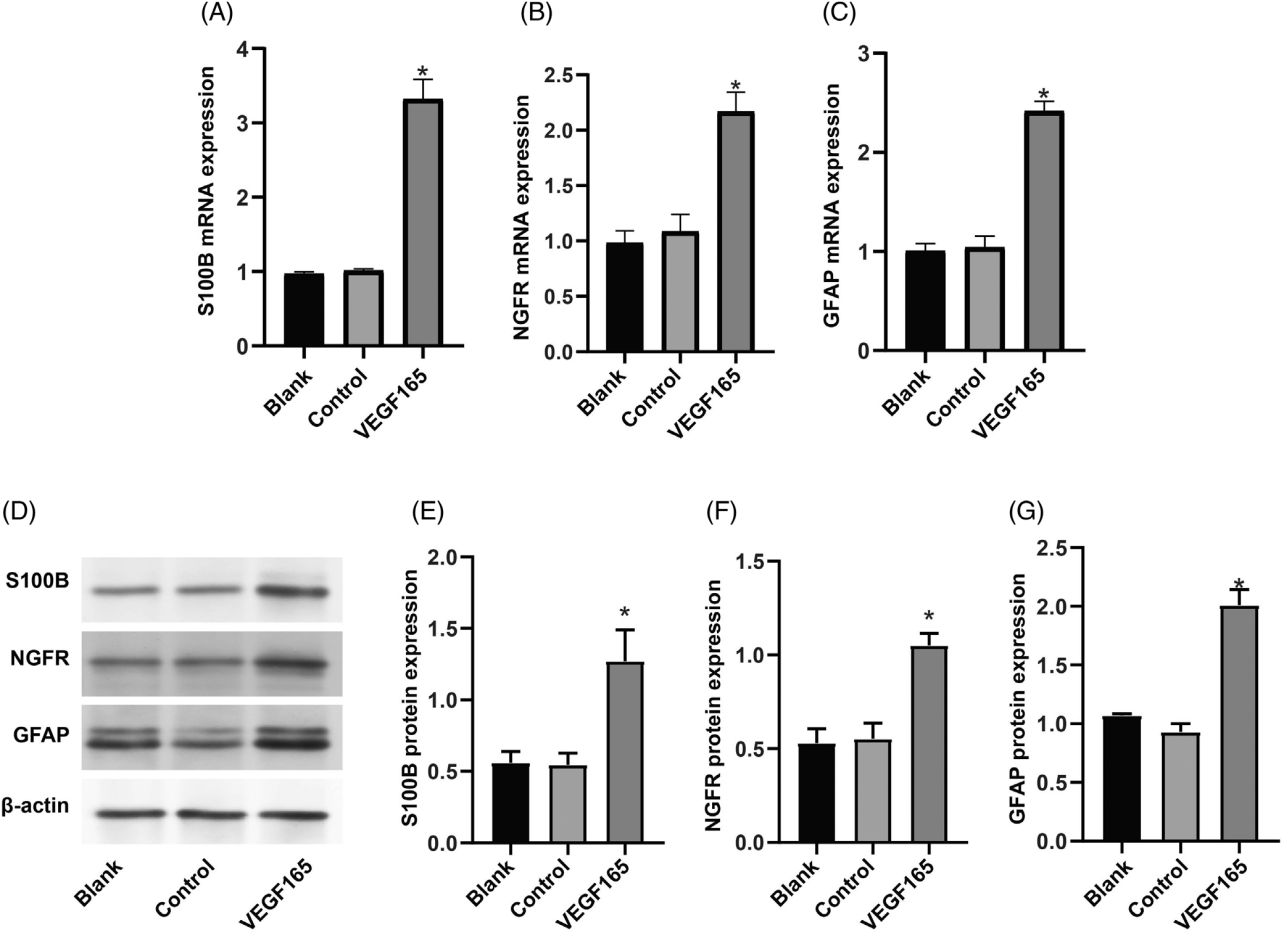
Investigating Schwann cell‐related genes in hADSCs following rAAV‐mediated VEGF165 modification and 14‐day culture in Schwann cell medium. Analysis of S100B, NGFR, and GFAP expressions in differentiated hADSCs following rAAV‐mediated VEGF165 modification and 14‐day culture in Schwann cell medium. Quantitative real‐time polymerase chain reaction (qRT‐PCR, A–C) and Western blotting (D–G) were performed. The study groups include the blank group (without infection), control group (rAAV control infection), and VEGF165 group (rAAV VEGF165 infection). Data are presented as mean ± SD of three independent experiments. * indicates significance compared to the blank and control groups at *p* < 0.05.

GFP‐positive ADSCs (GFP‐ADSCs) were detected as early as day 2 following viral infection, indicating a robust infection efficiency of ~90% as observed under microscopic examination (Figure 1A). The MTT assay revealed a trend of increased cell viability of hADSCs with rAAV VEGF165 vector, although statistical significance was not reached (all *p* > 0.05, Figure 1B). Morphologically, hADSCs undergoing Schwann cell induction displayed distinctive changes, with the VEGF165 group exhibiting more pronounced Schwann cell‐like features than the blank group and control group (Figure 1C). Substantial differences in VEGF gene and protein expressions were evident between undifferentiated hADSCs and differentiated Schwann cells, with markedly elevated levels observed in the latter (all *p <* 0.05, Figure 1D–F). Moreover, following rAAV‐VEGF165 transduction, both VEGF165 gene and protein expressions significantly increased compared to the control group (both *p <* 0.05). Moreover, rAAV‐mediated modifications of hADSCs cultured in Schwann cell medium for 14 days markedly enhanced Schwann cell‐related gene and protein expression (S100B, NGFR, and GFAP), as demonstrated by qRT‐PCR (Figure 2A–C) and Western blotting (Figure 2D–G) (all *p <* 0.05).

### 3.2 The hADSCs modified with rAAV‐VEGF165 enhances motor function and nerve regeneration in a sciatic nerve injury model

**FIGURE 3:**
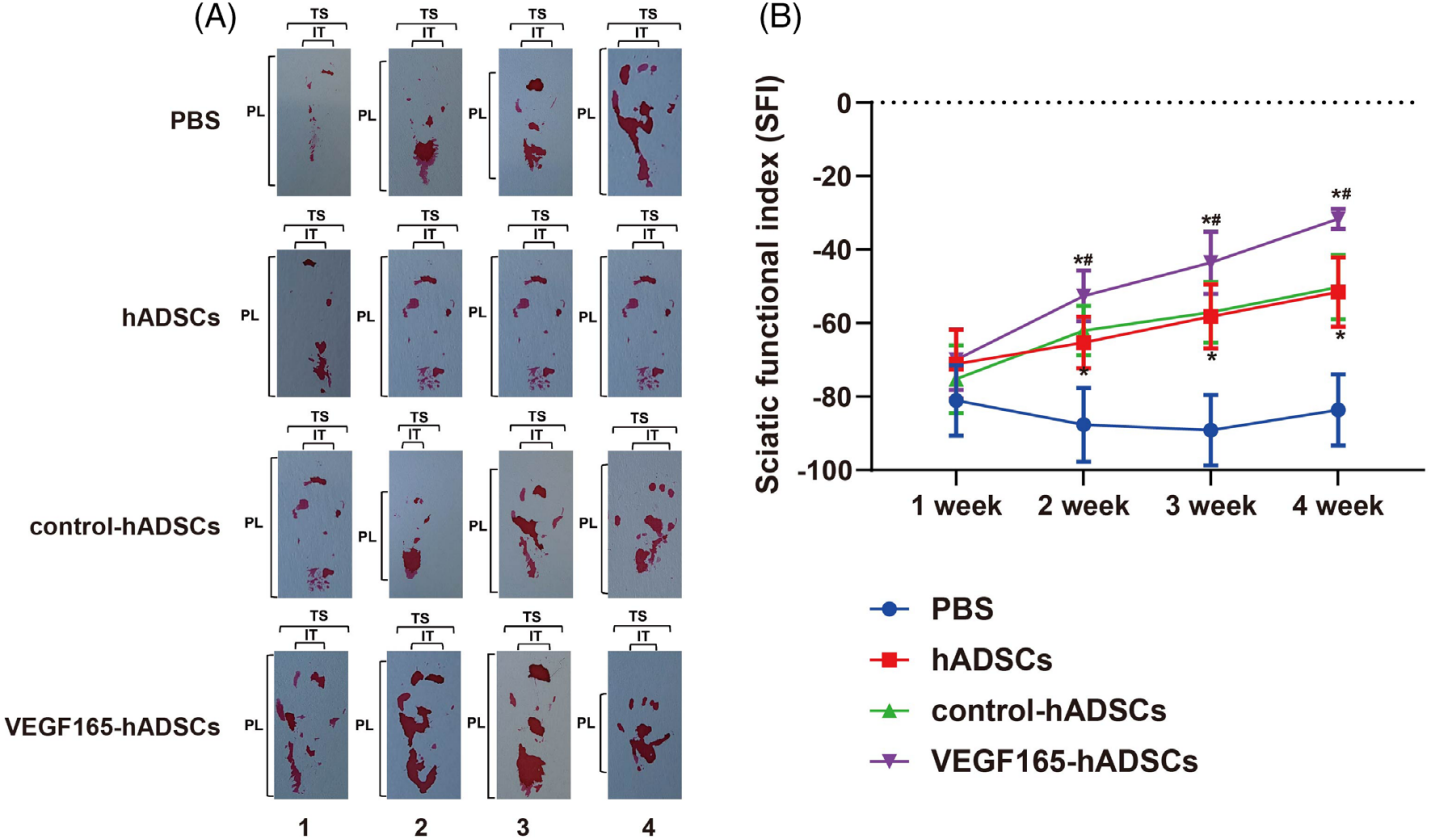
Impact of recombinant adeno‐associated virus (rAAV)‐mediated VEGF165 in hADSCs on motor function in a sciatic nerve injury model. (A) Footprint analysis assessed gait dynamics from 1 to 4 weeks post‐surgery, using hind paws coated in red ink to measure print length (PL), toe spread (TS), and intermediary toe spread (IT). (B) Sciatic function index (SFI) analysis. * indicates significance compared to the PBS group. # indicates significance compared to the hADSCs and control‐hADSCs groups. *n* = 6 animals/group.

**FIGURE 4:**
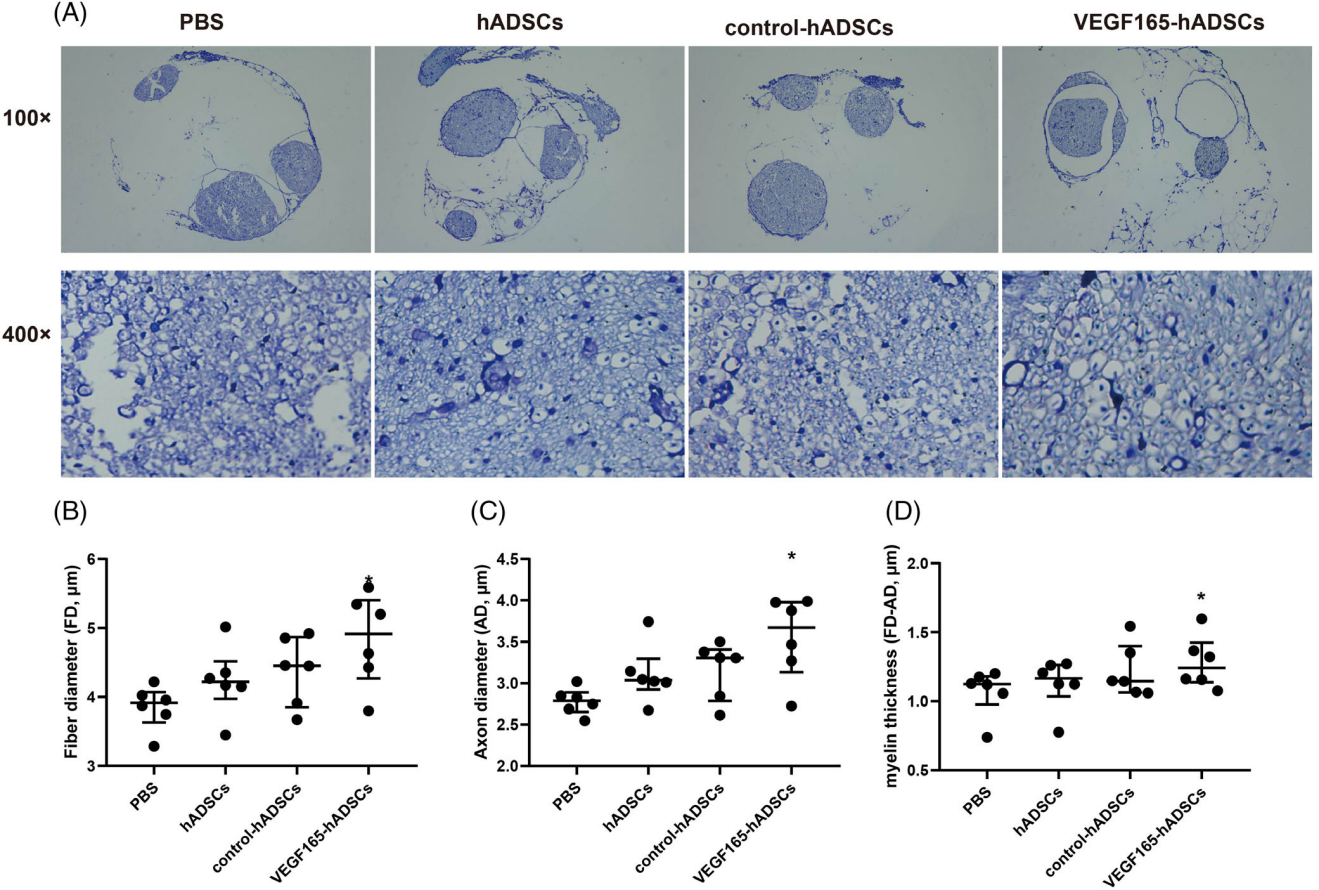
Impact of recombinant adeno‐associated virus (rAAV)‐mediated VEGF165 in hADSCs on nerve regeneration in a sciatic nerve injury model. (A) Histological examination utilizing toluidine blue staining. (B) Morphometric analysis of fiber diameter (FD), axon diameter (AD), and myelin thickness. * indicates significant difference compared to the PBS group (*p* < 0.05). # indicates significant difference compared to the hADSCs and control‐hADSCs groups (*p* < 0.05). *n* = 6 animals/group.

Motor function assessment across four treatment groups (PBS, hADSCs, control‐hADSCs, and VEGF165‐hADSCs) revealed no significant differences at 1 week (all *p >* 0.05). However, at 2, 3, and 4 weeks, the hADSCs and control‐hADSCs groups consistently demonstrated superior outcomes compared to the PBS group (all *p <* 0.001). Notably, the VEGF165‐hADSCs group exhibited substantial and consistent advantages in motor function improvement over hADSCs and control‐hADSCs groups (all *p <* 0.001) at 2, 3, and 4 weeks (Figure 3). Toluidine blue staining showed disorganized nerve fiber arrangement in the PBS group, while the hADSCs and control‐hADSCs groups exhibited relatively orderly nerve fiber alignment. The VEGF165‐hADSCs group displayed well‐organized and distinct nerve fiber morphology (Figure 4A). Morphometric analysis (Figure 4B–D) demonstrated significantly greater FD, AD, and myelin thickness in the VEGF165‐hADSCs group compared to the other three groups (all *p <* 0.05).

### 3.3 Enhanced expression of SC markers and neurotrophic factors in distal nerve segment following VEGF165‐hADSCs injection at 4 weeks post‐surgery

**FIGURE 5:**
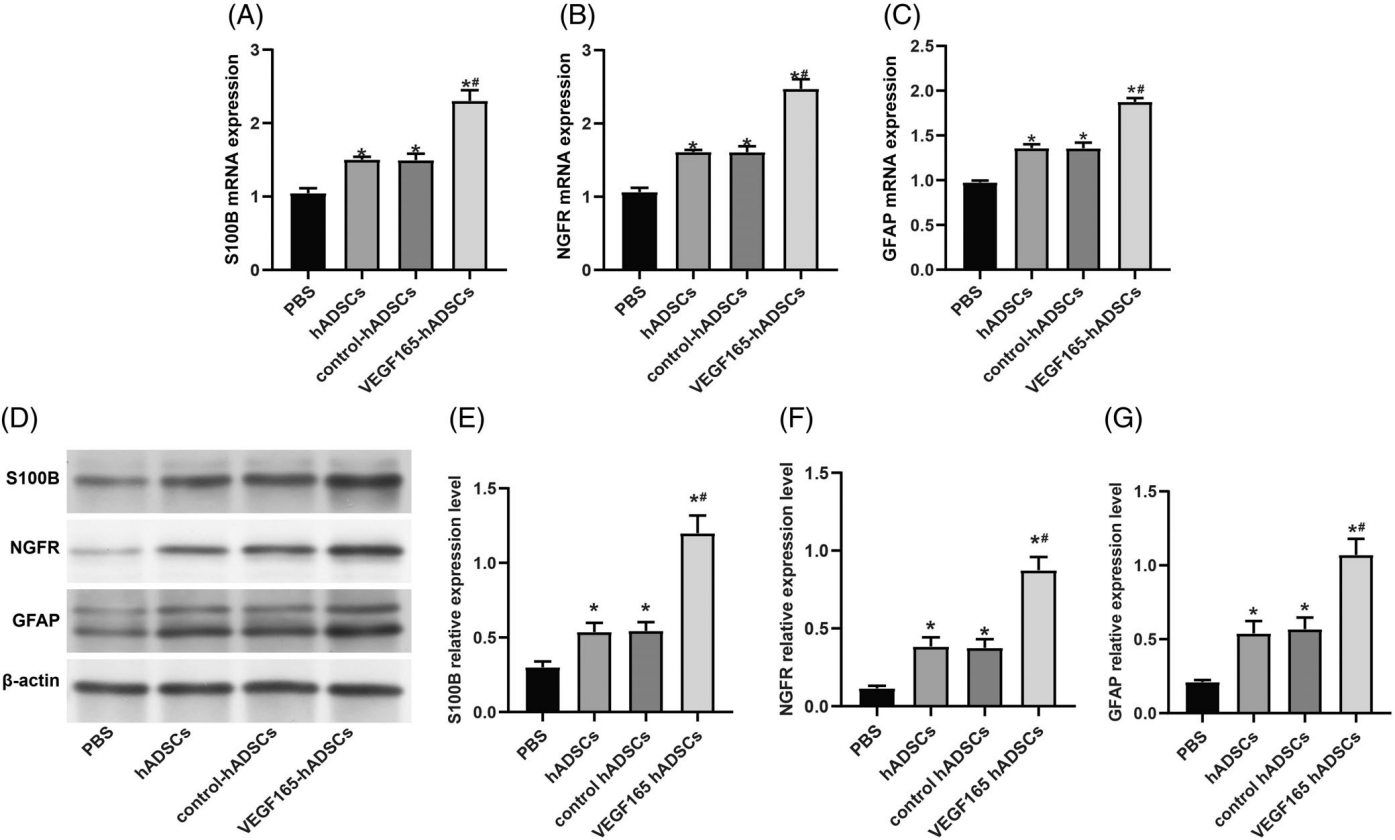
VEGF165‐hADSCs injection elevated expression of Schwann cell markers in the distal nerve segment at 4 weeks post‐surgery. (A–C) Quantitative real‐time polymerase chain reaction (qRT‐PCR) analysis of S100 calcium‐binding protein B (S100B), nerve growth factor receptor (NGFR), and glial fibrillary acidic protein (GFAP) gene expression. (D–G) Western blotting assessment of S100B, NGFR, and GFAP protein expression. * indicates significant difference compared to the PBS group (*p* < 0.05); # indicates significant difference compared to the hADSCs and control‐hADSCs groups (*p* < 0.05). *n* = 6 animals/group.

**FIGURE 6:**
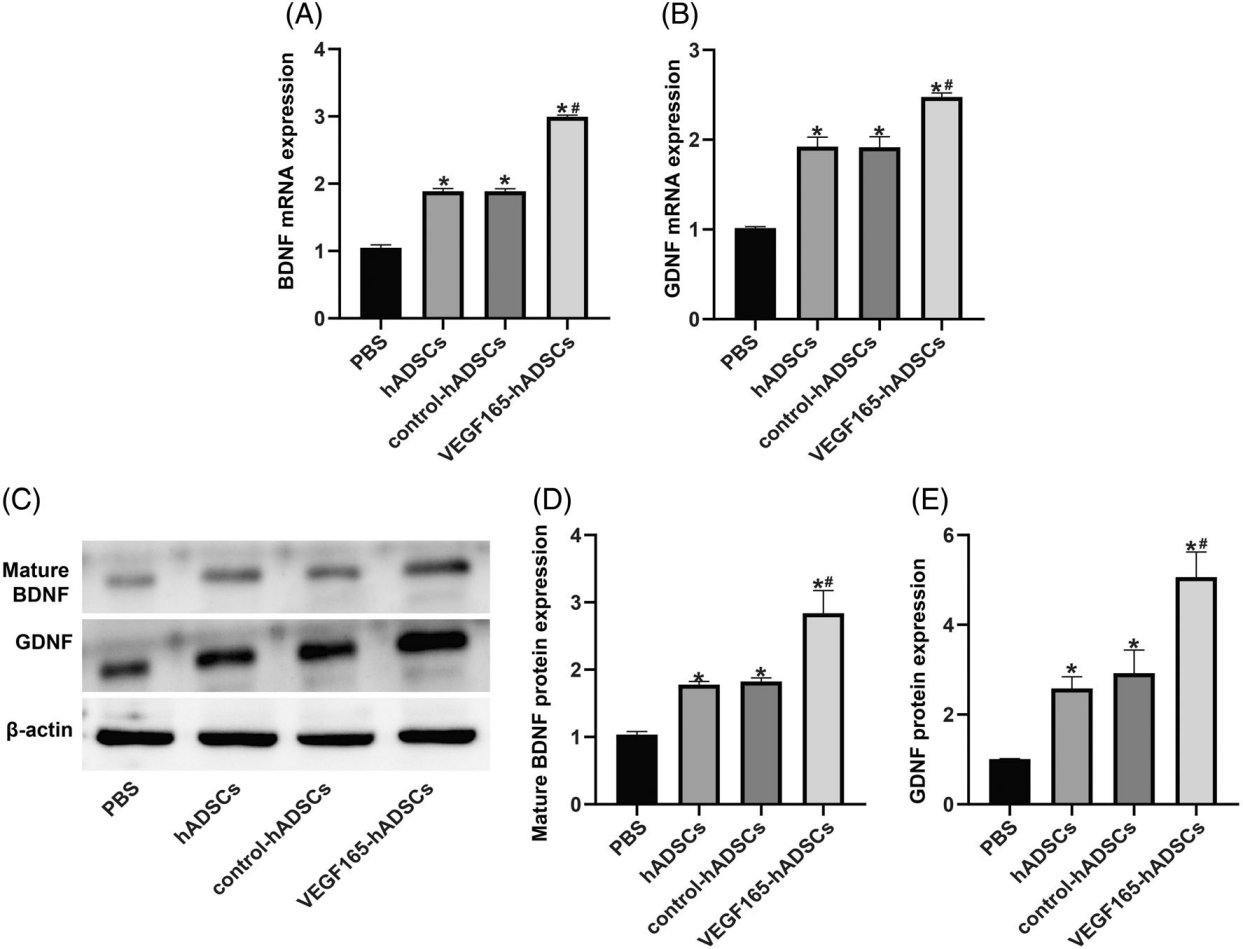
(A,B) Quantitative real‐time polymerase chain reaction (qRT‐PCR) analysis of brain‐derived neurotrophic factor (BDNF) and glial cell‐derived neurotrophic factor (GDNF) gene expression. (C–E) Western blotting assessment of mature BDNF and GDNF protein expression. * indicates significant difference compared to the PBS group (*p* < 0.05); # indicates significant difference compared to the hADSCs and control‐hADSCs groups (*p* < 0.05). *n* = 6 animals/group.

At the 4‐week postoperative stage, evaluation of Schwann cell markers (Figure 5) and neurotrophic factors (Figure 6) expression in the distal nerve segment was conducted using qRT‐PCR and Western blotting. The findings indicated a significant enhancement in the expression of S100B, NGFR, GFAP, mature BDNF, and GDNF within the distal nerve segment following hADSCs injection (all *p <* 0.05). Notably, the VEGF165‐ADSCs group exhibited a more pronounced upregulation compared to the hADSCs and control‐hADSCs groups (all *p <* 0.05).

## 4 DISCUSSION

PNI presents a formidable challenge in clinical practice, necessitating innovative therapeutic strategies to enhance functional recovery. While autologous nerve anastomosis remains the gold standard, achieving optimal outcomes remains elusive. This study delves into the potential of hADSCs modified with a rAAV carrying the VEGF165 gene in the context of PNI.

Recent studies have unveiled the ability of the VEGF165 gene to modulate stem cells in various diseases. For instance, gene‐modified human umbilical cord blood MSCs expressing VEGF165 have demonstrated protective effects against acute liver failure in rats.^21^ In the context of osteogenesis, VEGF165 transfection into bone marrow stromal stem cells has shown a slight reduction in osteogenic effects compared to controls at 21 days, suggesting a regulatory role in bone marrow stromal cell differentiation.^22^ Furthermore, VEGF165 adenoviral vector‐mediated transfection has promoted sustained expression of the target protein in hADSCs, emphasizing its potential in enhancing hADSCs proliferation for tissue defect treatment.^23^ In cardiac applications, myocardial MSCs injected with a hVEGF165 plasmid have shown improved cardiac function, possibly through the down‐regulation of myocardial TGF‐β1 expression and a reduction in the type I/III collagen ratio in a dilated cardiomyopathy rat model.^24^ In addition, when transfected by the VEGF165 gene, the BMSCs of a rat can better promote the regeneration of capillaries, which can improve the survival rate of transplanted free‐fat tissue.^25^

In the current study, we utilized hADSCs and VEGF165‐hADSCs at the third generation, supported by previous findings. Studies employing AAV‐VEGF165 gene‐modified hADSCs at passage 3 demonstrated potent therapeutic effects in ischemic skeletal muscle, suggesting potential for clinical angiogenesis.^17^ Additionally, recent research highlighted the effectiveness of VEGF165 modified mRNA (modVEGF165)‐engineered hADSCs at passage 3 for soft tissue reconstruction and rejuvenation.^26^ The rAAV‐mediated VEGF165 modification enhances the potential of hADSCs in the context of PNI by promoting Schwann cell differentiation. Schwann cells play a crucial role in nerve regeneration, and their ability to transition to a repair‐competent state significantly contributes to the intricate process of regeneration following PNI.^27^ In vitro analyses on primary Schwann cell cultures have revealed increased migration following stimulation with VEGF165, underscoring its positive impact on glial cell migration—an essential process in peripheral nerve regeneration.^16^ Schwann cell markers, including S100B, NGFR, and GFAP, serve as critical indicators in identifying and characterizing Schwann cells in the peripheral nervous system.^28^ The observed elevation in the expression of these markers in VEGF165‐modified hADSCs highlights their enhanced differentiation potential, aligning with existing literature emphasizing the crucial role of Schwann cells in supporting nerve regeneration.^29^ The observed elevated expression of Schwann cell markers (S100B, NGFR, and GFAP) in VEGF165‐modified hADSCs highlights their enhanced differentiation potential. This finding aligns with existing literature emphasizing the crucial role of Schwann cells in supporting nerve regeneration.^30^

Notably, adenoviral gene transfer of VEGF165 has demonstrated significant therapeutic impacts on postpartum brachial plexus nerve injury in rats. This intervention promoted faster nerve regeneration, reduced degeneration and mass loss in the deltoid muscle, and enhanced the survival of motoneurons, affirming the positive regenerative and neuroprotective effects of VEGF165 gene therapy on nerve cells.^31^ VEGF‐165 treatment in a rat peripheral nerve injury model enhanced axonal sprouting and increased axon quantity and neural tissue percentage at the proximal nerve graft coaptation site, leading to improved reinnervation and greater muscle masses.^32^ In our research, in vivo experiments using a sciatic nerve injury mouse model have shown promising outcomes. Mice receiving VEGF165‐modified hADSCs exhibited superior motor function recovery compared to other groups. Quantitative footprint analysis provided evidence of enhanced motor function, establishing the therapeutic efficacy of VEGF165‐modified hADSCs. Histological assessments further supported these findings, revealing improved nerve fiber morphology, increased myelinated fiber area, myelin thickness, and axonal area in the VEGF165‐hADSCs group.

BDNF, synthesized initially as pro‐BDNF, undergoes cleavage to form mature BDNF, which activates TrkB receptors, influencing neural circuit function and behavior.^33^ During PNI, there is a notable increase in mature BDNF expression, primarily sourced from Schwann cells or mast cells.^34^ Enhanced mature BDNF expression in activated Schwann cells facilitates peripheral nerve regeneration through various mechanisms, including regulation of calcium channel proteins, boosting antioxidant enzyme activity, and modulating neuronal gene expression.^35^ In contrast, pro‐BDNF, acting via the p75 receptor, negatively regulates hippocampal dendritic complexity and spine density, leading to depressed synaptic activity and enhanced long‐term depression.^36^ Our focus on mature BDNF aligns with its extensive research due to its pivotal role in PNI.^37^ GDNF emerges as a promising treatment candidate due to its involvement in neuronal differentiation and its recognition as a potent motoneuron survival and axon outgrowth factor.^38^ GDNF‐mediated signaling cascades significantly contribute to myelination, proliferation, and migration in Schwann cells.^39^ Exogenous GDNF application enables Schwann cells to overcome phenotypic mismatch‐induced growth inhibition, inducing motor neuron axonal growth.^40^ The heightened expression of neurotrophic factors, including mature BDNF and GDNF, in the distal nerve segment further substantiates the contribution of VEGF165‐modified hADSCs to creating a conducive microenvironment for nerve regeneration.

While our study offers promising avenues for PNI treatment, it is crucial to acknowledge limitations and areas for future research. One limitation is the limited exploration of varying cell numbers of VEGF165‐hADSCs on SNI recovery due to time and funding constraints. Future research should prioritize investigating this aspect to refine treatment understanding and clinical applicability. In addition, a thorough investigation into the long‐term safety profile of VEGF165‐modified hADSCs, including the risk of tumorigenesis, is necessary. Understanding optimal dosage and delivery methods is crucial for translating findings into effective therapeutic interventions.

In conclusion, this study demonstrates that rAAV‐VEGF165 modification enhances the therapeutic potential of hADSCs for PNI. The observed improvements in Schwann cell differentiation, motor function recovery, and nerve regeneration provide a solid foundation for further exploration of this innovative approach in the clinical context. However, careful consideration of safety aspects and optimization of therapeutic parameters is crucial for the successful translation of these findings into clinical practice.

## CONFLICT OF INTEREST STATEMENT

The authors declare that they have no competing interests.

## Supporting information


**Data S1** Supporting Information.
