# Supplementary material for: Enhancing therapeutic potential: Human adipose‐derived mesenchymal stem cells modified with recombinant adeno‐associated virus expressing VEGF165 gene for peripheral nerve injury
Source: Kaohsiung J Med Sci. 2024 Aug 5;40(9):819–29. doi: 10.1002/kjm2.12875 (PMC11895634; doi:10.1002/kjm2.12875)
Supplement: Supplementary file 1 — Data S1 Supporting Information. [file KJM2-40-819-s001.docx]

"Original full-length membrane images of Western blot."
